# Radiomics Facilitates Candidate Selection for Irradiation Stents Among Patients With Unresectable Pancreatic Cancer

**DOI:** 10.3389/fonc.2019.00973

**Published:** 2019-09-27

**Authors:** Hai-Feng Zhou, Yu-Qi Han, Jian Lu, Jing-Wei Wei, Jin-He Guo, Hai-Dong Zhu, Ming Huang, Jian-Song Ji, Wei-Fu Lv, Li Chen, Guang-Yu Zhu, Zhi-Cheng Jin, Jie Tian, Gao-Jun Teng

**Affiliations:** ^1^Center of Interventional Radiology and Vascular Surgery, Department of Radiology, Zhongda Hospital, Medical School, Southeast University, Nanjing, China; ^2^School of Life Science and Technology, Xidian University, Xi'an, China; ^3^Key Laboratory of Molecular Imaging, Institute of Automation, Chinese Academy of Sciences, Beijing, China; ^4^University of Chinese Academy of Sciences, Beijing, China; ^5^Department of Minimally Invasive Interventional Radiology, Yunnan Tumor Hospital, The Third Affiliated Hospital of Kunming Medical University, Kunming, China; ^6^Department of Radiology, Lishui Central Hospital, Wenzhou Medical University, Lishui, China; ^7^Department of Interventional Radiology, Anhui Provincial Hospital, The First Affiliated Hospital of University of Science and Technology of China, Hefei, China; ^8^Beijing Advanced Innovation Centre for Big Data-Based Precision Medicine, School of Medicine, Beihang University, Beijing, China; ^9^Engineering Research Centre of Molecular and Neuro Imaging of Ministry of Education, School of Life Science and Technology, Xidian University, Xi'an, China

**Keywords:** radiomics, pancreatic cancer, malignant biliary obstruction, irradiation stent, survival

## Abstract

**Purpose:** To develop a model to select appropriate candidates for irradiation stent placement among patients with unresectable pancreatic cancer with malignant biliary obstruction (UPC-MBO).

**Methods:** This retrospective study included 106 patients treated with an irradiation stent for UPC-MBO. These patients were randomly divided into a training group (74 patients) and a validation group (32 patients). A clinical model for predicting restenosis-free survival (RFS) was developed with clinical predictors selected by univariate and multivariate analyses. After integrating the radiomics signature, a combined model was constructed to predict RFS. The predictive performance was evaluated with the concordance index (C-index) in both the training and validation groups. The median risk score of progression in the training group was used to divide patients into high- and low-risk subgroups.

**Results:** Radiomics features were integrated with clinical predictors to develop a combined model. The predictive performance was better in the combined model (C-index, 0.791 and 0.779 in the training and validation groups, respectively) than in the clinical model (C-index, 0.673 and 0.667 in the training and validation groups, respectively). According to the median risk score of 1.264, the RFS was significantly different between the high- and low-risk groups (*p* < 0.001 for the training group, and *p* = 0.016 for the validation group).

**Conclusions:** The radiomics-based model had good performance for RFS prediction in patients with UPC-MBO who received an irradiation stent. Patients with slow progression should consider undergoing irradiation stent placement for a longer RFS.

## Introduction

Pancreatic cancer is one of the leading causes of cancer-related death (1), and it has the lowest five-year relative survival rate among those with any type of cancer (approximately 8% for all stages) (2). Less than 20% of patients with pancreatic cancer are candidates for surgical resection (3, 4), and over half of them develop obstructive jaundice (5). Considering that patients with advanced pancreatic cancer have only a 6–10 month median survival, the general treatment is palliative care (6). Chemotherapy, radiotherapy, targeted therapy and immunotherapy are not always used for unresectable pancreatic cancer (UPC) patients due to poor performance status, limited effects and added toxicity (3, 7). Placement of a self-expanding metal stent is the standard palliative care for UPC patients with malignant biliary obstruction (MBO) (8–10). Intraluminal irradiation stents, which combined a self-expanding metal stent with brachytherapy to treat local obstructive lesions, were demonstrated to have better patency and be associated with longer survival than conventional stents (uncovered self-expanding metal stents) for unresectable MBO (11, 12). Although the subgroup analysis of overall survival according to tumor etiology showed better survival for biliary tract cancer, there did not appear to be a significant difference in patients with pancreatic cancer (12). Therefore, it is important to select appropriate candidates with pancreatic cancer to undergo irradiation stent placement, not only for individual and reasonable stent selection, but also for prolonged patency and improved survival.

Currently, different models have been developed to predict survival outcomes in patients with different stages of pancreatic cancer (13–16). A consensus statement also proposed clinical prognostic variables for UPC (17). Moreover, imaging-based or radiomic biomarkers have been reported to be available for the prognostic prediction of patients with pancreatic cancer, based on computed tomography (CT) (18–23), magnetic resonance imaging (24, 25), positron-emission tomography (25–27) and fluorescence microscopic imaging (28) findings. Radiomics, a novel method of in-depth feature analysis, is to quantify and extract the high-throughput imaging features from radiographic images (20). Radiomics, such as texture analysis, reflects different imaging phenotypes and tumor heterogeneity, which can be used to assess survival outcomes and predict treatment response (19, 21, 27). However, there are no tools to predict the survival benefits from irradiation stent placement in patients with UPC-MBO. A predictive model based on clinical and imaging features will offer an objective, convenient and non-invasive method for determining appropriate treatment options and making better clinical decisions, especially critical decisions in patients with UPC-MBO.

In this study, we proposed a novel model incorporating clinical biomarkers and CT radiomics features to predict restenosis-free survival (RFS) for individual patients with UPC-MBO who undergoing irradiation biliary stent placement. According to our proposed model, irradiation stent placement could be recommended for appropriate candidates with slow progression for a longer RFS.

## Materials and Methods

This multicenter retrospective study was approved by the institutional review boards at all participating centers. The need for informed consent was waived due to the study's retrospective nature.

### Patients

**Figure 1:**
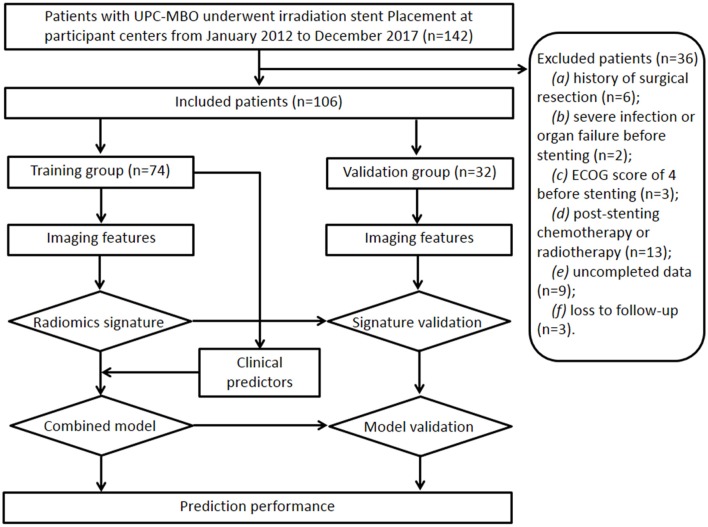
Flowchart of the study design and patient exclusion.

Between January 2012 and December 2017, 106 patients (69 males, 37 females; mean age, 66 ± 12 years [standard deviation]; age range, 40–86 years) treated with irradiation stent placement for UPC-MBO from four centers were finally included and randomly divided into a training group (74 patients) and a validation group (32 patients). The sample size calculation is shown in Appendix E1. The study design and patient exclusion criteria are illustrated in Figure 1.

The inclusion criteria were as follows: (a) age 18 years or older; (b) clinical or histopathological diagnosis of UPC-MBO; (c) unresectable disease due to extensive lesions, metastases, a poor medical condition, or refusal to undergo surgery; (d) initial percutaneous transhepatic biliary stent placement; and (e) standard contrast-enhanced CT performed <2 weeks before stenting. The exclusion criteria were as follows: (a) history of surgical resection, (b) presence of severe infection or organ failure before stenting, (c) Eastern Cooperative Oncology Group score of 4 before stenting, (d) any other anticancer therapy except supportive treatment after stenting, (e) incomplete clinical or CT imaging data, or (f) loss to follow-up.

The following clinical characteristics were recorded: (a) demographics, including sex, age, and body mass index; (b) preprocedural status, including pain as assessed by a visual analog scale score, Eastern Cooperative Oncology Group performance status, prior biliary drainage, history of chemotherapy, history of radiotherapy, and degree of ascites; (c) preprocedural blood biochemical analysis, including total bilirubin, direct bilirubin, direct bilirubin/total bilirubin ratio, carbohydrate antigen (CA) 19-9, CA125, and carcinoembryonic antigen; and (d) parameters related to pancreatic cancer, such as the tumor stage according to the TNM classification system (American Joint Committee on Cancer, 8th ed., 2017) (29), liver metastasis, number of metastatic lesions, and length of obstruction.

A standard percutaneous transhepatic biliary stenting procedure was performed under fluoroscopic guidance with or without ultrasonographic guidance by interventional radiologists with more than 15 years of experience. The irradiation stent consisted of two overlapping parts, an outer 125I seed-loaded stent and an inner conventional uncovered self-expanding metal stent (Nanjing Micro-Tech Co., Ltd., Nanjing, China). The two parts were assembled in the biliary tract during the procedure. The 125I seeds (CIAE-6711; Chinese Atomic Energy Science Institution, Beijing, China) were preloaded into the sheaths that were attached to the outer surface of the stent immediately before the procedure. According to the Treatment Planning System (TPS, FTT Technology Ltd. Co., Beijing, China), the number, dosage, and distribution of the 125I seeds were calculated. The standards of radiation safety and management were performed after irradiation stent placement (30).

Routine follow-up, including performance status, clinical signs, postprocedural treatment, blood biochemical analysis, and imaging, was performed 1 week after stent placement, monthly for 6 months, and then every 3 months. The endpoint of this study was the occurrence of restenosis or death. RFS was calculated from the date of stenting to the date of the endpoint, which equaled the duration of stent function. Restenosis indicated stent dysfunction, which was defined by clinical signs of recurrent jaundice with elevated bilirubin levels along with biliary reobstruction as evidenced on CT, ultrasound, magnetic resonance cholangiopancreatography, or percutaneous transhepatic cholangiography. RFS was censored at the date of the last follow-up visit for restenosis-free patients.

### CT Image Acquisition and Tumor Segmentation

Imaging feature extraction was performed on each patient's CT images within 2 weeks before stent placement. The pancreatic CT scan included an arterial phase and a portal-venous phase, which were used to extract imaging features. The CT acquisition protocols and image preprocessing were described in Appendix E2. The region of interest (ROI) was drawn with ITK-SNAP software (version 3.4.0; www.itksnap.org) by an experienced radiologist (reader 1). Each two-dimensional CT image covering the visible tumor region was delineated along the tumor boundaries, and the overlap in the delineated areas was selected as the final ROI (Figure E1). The ROIs for the arterial and venous phases were annotated. The stability of radiomics features was verified from the ROI regions that were annotated by two radiologists (reader 1 and reader 2) separately through intra- and interobserver correlation coefficients. Correlation coefficients ranging from 0 to 1 were considered, and values > 0.8 were considered almost perfect agreement (31).

### Imaging Feature Extraction

Radiomics features were extracted from the ROIs using MATLAB (version R2018a; Mathworks; Natick, USA), including 25 non-texture features, 51 texture features and wavelet features in wavelet images decomposed on different scales. Non-texture features reflect the shape, size and intensity of tumor lesions, and texture features represent the inherent heterogeneity of tumors based on four textural matrices. In addition, a three-dimensional wavelet transform was applied to decouple the first-order statistical features and texture features for each CT image. Finally, we extracted 620 radiomics features from original CT images and wavelet decompositions in each phase from each patient. The details of these features are exhibited in Appendix E3 and Table E1.

### Image Feature Reduction and Radiomics Model Construction

For feature preselection, intra-, and inter-observer coefficients were used to detect the stability of features with a threshold of 0.8. Then, Pearson's correction analysis was applied to identify redundant and collinear features, and features with mutual correlation coefficients >0.9 were excluded. After initial selection, the least absolute shrinkage and selection operator (LASSO)-Cox regression approach was used to identify predictive factors for RFS in the training group (32). Ten-fold cross-validation was used to optimize the regression model to select the most reliable model. The minimum tuning parameter (lambda) was used in LASSO-Cox regression approach. A radiomics signature was constructed by a weighted linear combination of selected features in the arterial and portal-venous phases, separately. A radiomics model was constructed by both radiomics signatures of the two phases using the Cox proportional hazard regression method. The Harrel concordance index (C-index) was calculated to describe the performance of the radiomics model.

### Clinical and Combined Models

Clinical and combined models were also built for comparison with the radiomics model. Univariate and multivariate Cox proportional hazard analyses were applied to identify effective clinical predictors. Based on the Cox proportional hazard regression model, a clinical model was constructed with clinical predictors, and the combined model integrated clinical predictors and the radiomics signature. In the combined model, the radiomics signature was calculated as the Rad-score for quantification. The C-index of the clinical and combined models was also calculated to illustrate their performance. The 3-month RFS rate of the combined model was assessed through receiver operating characteristic curve analysis along with the area under the curve. Decision curve analysis was used to compare the net benefit at different threshold probabilities from the clinical and combined nomograms.

### Statistical Analysis

Continuous variable is described as mean ± standard deviation, and categorical variable is described as number and percentage. Baseline characteristics between two groups were compared by Student's *t*-test for continuous variables and by Pearson's chi squared or Fisher's exact test for categorical variables. With the R package (version 3.4.4; R Package for Statistical Computing; www.r-project.org), the nomograms were formulated in the training group based on the results of the multivariate analysis and by the Cox proportional hazard regression modeling strategies. Receiver operating characteristic curves were drawn and the area under the curve was calculated to evaluate the discrimination performance for 3-month RFS. Calibration curves were drawn to compare the 3-month RFS between the predicted and actual outcomes using the Hosmer–Lemeshow test. Decision curve analysis was used to evaluate the clinical utility of the nomogram by calculating the net benefit at different threshold probabilities. The combined model generated a risk score for RFS and dichotomized the patients into two groups with different risks of progression using the median risk score in the training group. Kaplan-Meier curves were generated to evaluate the ability of the risk score to stratify the patients, and log-rank tests were applied to assess the statistical significance with *p* < 0.05.

## Results

### Patients

A total of 106 patients (69 males, 37 females; mean age, 66 ± 12 years [standard deviation]; age range, 40–86 years) were included in this study, including 74 patients in the training group and 32 patients in the validation group. The clinical characteristics showed no significant differences between the two groups (all *p* > 0.05, Table 1). During the mean follow-up time of 165.3 days, 99 of 106 (93%) patients reached the endpoint. There was no significant difference in the median RFS between the training group (139.5 days) and the validation group (120 days) (*p* = 0.926).

**Table 1:** Patient characteristics in the training and validation groups.

**Characteristics**	**Total**	**Training**	**Validation**	***p*-value**
	**(*n* = 106)**	**(*n* = 74)**	**(*n* = 32)**	
Age, mean ± SD, years	65.63 ± 11.95	66.41 ± 12.27	63.84 ± 11.71	0.313
**Sex, n (%)**				0.713
Male	69 (65.1)	49 (66.2)	20 (62.5)	
Female	37 (34.9)	25 (33.8)	12 (37.5)	
BMI, mean ± SD, kg/m^2^	20.59 ± 3.07	20.39 ± 3.12	21.05 ± 2.94	0.312
Length of obstruction, mean ± SD, mm	37.67 ± 10.03	37.61 ± 9.91	37.81 ± 10.47	0.924
TB, mean ± SD, μmol/L	185.09 ± 134.44	179.60 ± 137.04	197.78 ± 129.45	0.525
DB, mean ± SD, μmol/L	139.33 ± 97.67	135.80 ± 99.51	147.48 ± 94.32	0.574
DB/TB ratio, mean ± SD	0.758 ± 0.110	0.756 ± 0.115	0.761 ± 0.100	0.829
**Pain, n (%)**				0.250
None	23 (21.7)	19 (25.7)	4 (12.5)	
Mild	63 (59.4)	43 (58.1)	20 (62.5)	
Moderate or severe	20 (18.9)	12 (16.2)	8 (25)	
**T stage, n (%)**				0.319
2	10 (9.4)	9 (12.2)	1 (3.1)	
3	11 (10.4)	8 (10.8)	3 (9.4)	
4	85 (80.2)	57 (77)	28 (87.5)	
**N stage, n (%)**				0.255
0	26 (24.5)	15 (20.3)	11 (34.4)	
1	68 (64.2)	51 (68.9)	17 (53.1)	
2	12 (11.3)	8 (10.8)	4 (12.5)	
**M stage, n (%)**				0.051
0	68 (64.2)	52 (70.3)	16 (50.0)	
1	38 (35.8)	22 (29.7)	16 (50.0)	
**Liver metastasis, n (%)**				0.361
No	76 (71.7)	55 (74.3)	21 (65.6)	
Yes	30 (28.3)	19 (25.7)	11 (34.4)	
**Number of metastatic lesions, n (%)**				0.099
0	68 (64.2)	50 (67.6)	18 (56.3)	
1	12 (11.3)	10 (13.5)	2 (6.3)	
≥2	26 (24.5)	14 (18.9)	12 (37.5)	
**Ascites level, n (%)**				0.541
None	85 (80.2)	61 (82.4)	24 (75)	
Mild	14 (13.2)	8 (10.8)	6 (18.8)	
Moderate or severe	7 (6.6)	5 (6.8)	2 (6.3)	
**Radiotherapy, n (%)**				0.137
No	101 (95.3)	72 (97.3)	29 (90.6)	
Yes	5 (4.7)	2 (2.7)	3 (9.4)	
**Chemotherapy, n (%)**				0.775
No	91 (85.8)	64 (86.5)	27 (84.4)	
Yes	15 (14.2)	10 (13.5)	5 (15.6)	
**ECOG score, n (%)**				0.774
0	3 (2.8)	2 (2.7)	1 (3.1)	
1	11 (10.4)	9 (12.2)	2 (6.3)	
2	60 (56.6)	40 (54.1)	20 (62.5)	
3	32 (30.2)	23 (31.1)	9 (28.1)	
**Prior PTBD, n (%)**				0.219
No	31 (29.2)	19 (25.7)	12 (37.5)	
Yes	75 (70.8)	55 (74.3)	20 (62.5)	
**CA19-9, n (%)**				0.349
<1,000 U/ml	57 (53.8)	42 (56.8)	15 (46.9)	
≥1,000 U/ml	49 (46.2)	32 (43.2)	17 (53.1)	
**CA125, n (%)**				0.660
<35 U/ml	33 (31.1)	24 (32.4)	9 (28.1)	
≥35 U/ml	73 (68.9)	50 (67.6)	23 (71.9)	
**CEA, n (%)**				0.870
<5 ng/ml	41 (38.7)	29 (39.2)	12 (37.5)	
≥5 ng/ml	65 (61.3)	45 (60.8)	20 (62.5)	

*Continuous variable is described as mean ± SD, and categorical variable is described as number and percentage. Baseline characteristics between two groups were compared by Student's t-test for continuous variables and by Pearson's chi squared or Fisher's exact test for categorical variables. SD, standard deviation; BMI, body mass index; TB, total bilirubin; DB, direct bilirubin; ECOG, Eastern Cooperative Oncology Group; PTBD, percutaneous transhepatic biliary drainage; CA, carbohydrate antigen; CEA, carcinoembryonic antigen*.

### Radiomics Features

We extracted 620 features from the arterial and venous phases. After intra- and interobserver agreement analysis, 368 features from the arterial phase and 324 features from the portal-venous phase were retained for collinearity testing (Figure E2). A total of 61 features from the arterial phase and 49 features from the portal-venous phase were identified as independent after Pearson's correlation analysis (Table E2). The LASSO-Cox model identified that eight features from the arterial phase and six features from the portal-venous phase were most efficient for predicting RFS (Figure E3). The eight biomarkers from arterial phase were “glszm_LZHGE,” “fos_median,” “glszm_SZSE,” “glcm_inverse_variance,” “fos_minimum,” “glcm_IMC2,” “glszm_LGLZE,” and “glszm_HGLZE.” The six biomarkers from portal-venous phase were “glszm_ZSV,” “fos_uniformity,” “glrlm_SRHGLE,” “glcm_correlation,” “ngtdm_complexity,” and “glszm_HGLZE.” These radiomics biomarkers showed no significant difference between the training and validation groups (all *p* > 0.05, Table E3).

### Radiomics Model

Regarding the LASSO-Cox model, the C-index in the arterial phase was 0.735 and 0.719 for the training and validation groups, respectively; the C-index in the portal venous phase was 0.768 and 0.788 for the training and validation groups, respectively. The radiomics model, which was developed by integrating the radiomics signatures of both phases, yielded higher C-indices of 0.787 and 0.796 for the training and validation groups, respectively (Table 2).

**Table 2:** The C-indexes of clinical, radiomic, and combined models.

**Models**	**Training**		**Validation**	
	**C-index**	**95% CI**	**C-index**	**95% CI**
Clinical model	0.673	(0.594, 0.751)	0.667	(0.541, 0.793)
Arterial phase features	0.735	(0.559, 0.911)	0.719	(0.445, 0.994)
Portal-venous phase features	0.768	(0.523, 1)	0.788	(0.413, 1)
Radiomics signature	0.787	(0.542, 1)	0.796	(0.421, 1)
Combined model	0.791	(0.614, 0.967)	0.779	(0.504, 1)

*C-index, concordance index; CI, confidence interval*.

The arterial phase score for progression to the endpoint was calculated with the following formula.

$$\begin{array}{lll} \text{AP}\_\text{score} & = & exp\;(-\text{0.32651538+ 0.18816560}\\ \; & \; & \times \text{AP}\_\text{Coif1}\_\text{glszm}\_\text{LZHGE}-\text{0.01513133}\\ \; & \; & \times \text{AP}\_\text{Coif2}\_\text{fos}\_\text{median}-\text{0.05629135}\\ \; & \; & \times \text{AP}\_\text{Coif5}\_\text{glszm}\_\text{SZSE}-\text{0.03575725}\\ \; & \; & \times \text{AP}\_\text{Coif7}\_\text{glcm}\_\text{inverse}\_\text{variance+0.10324552}\\ \; & \; & \times \text{AP}\_\text{Coif8}\_\text{fos}\_\text{minimum}-\text{0.06760264}\\ \; & \; & \times \text{AP}\_\text{Coif8}\_\text{glcm}\_\text{IMC2}-\text{0.22867284}\\ \; & \; & \times \text{AP}\_\text{Coif8}\_\text{glszm}\_\text{LGLZE+0.03670083}\\ \; & \; & \times \text{AP}\_\text{Coif8}\_\text{glszm}\_\text{HGLZE}) \end{array}$$

The portal-venous phase score for progression to the endpoint was calculated with the following formula.

$$\begin{array}{lll} \text{PP}\_\text{score} & = & exp\;(-\text{0.29073968+0.14320514}\\ \; & \; & \times \text{PP}\_\text{ori}\_\text{glszm}\_\text{ZSV+0.05375225}\\ \; & \; & \times \text{PP}\_\text{Coif1}\_\text{fos}\_\text{uniformity}-\text{0.26501636}\\ \; & \; & \times \text{PP}\_\text{Coif2}\_\text{glrlm}\_\text{SRHGLE}-\text{0.03923173}\\ \; & \; & \times \text{PP}\_\text{Coif5}\_\text{glcm}\_\text{correlation}-\text{0.01452813}\\ \; & \; & \times \text{PP}\_\text{Coif6}\_\text{ngtdm}\_\text{complexity+0.01951407}\\ \; & \; & \times \text{PP}\_\text{Coif8}\_\text{glszm}\_\text{HGLZE} \end{array}$$

The total radiomics score for progression to the endpoint was calculated with the following formula.

$$\begin{array}{l} \text{Rad-score}=exp\;\left(0.463\times \text{AP\_score}+0.665\times \text{PP\_score}\right)\; \end{array}$$

### Clinical and Combined Models

**Figure 2:**
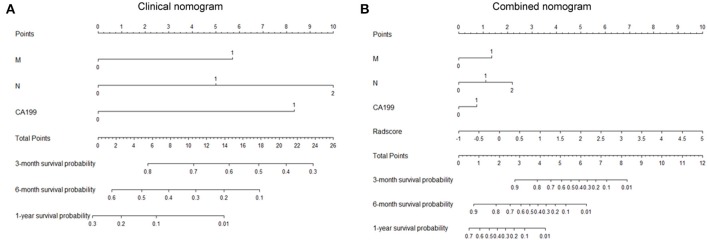
Nomograms for the clinical and combined models. **(A)** Clinical nomogram based on three clinical predictors. **(B)** Combined nomogram based on three clinical predictors and the radiomics signature. To use these nomograms, the user locates an individual patient's value on each variable axis and draws a line up to determine the number of points received for each variable value. The sum of these numbers is located on the axis of total points, and three lines are drawn down to the risk axes to determine the 3-, 6-, and 12-month RFS probabilities.

**Figure 3:**
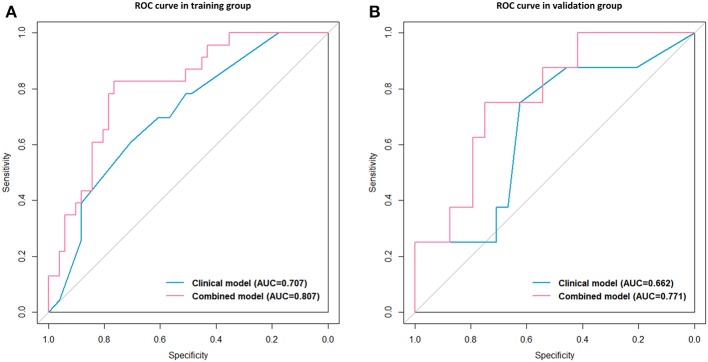
Receiver operating characteristic (ROC) curves with the area under the curve (AUC) for the predictive performance for 3-month RFS. Clinical model vs. combined model in the training group **(A)** and the validation group **(B)**.

**Figure 4:**
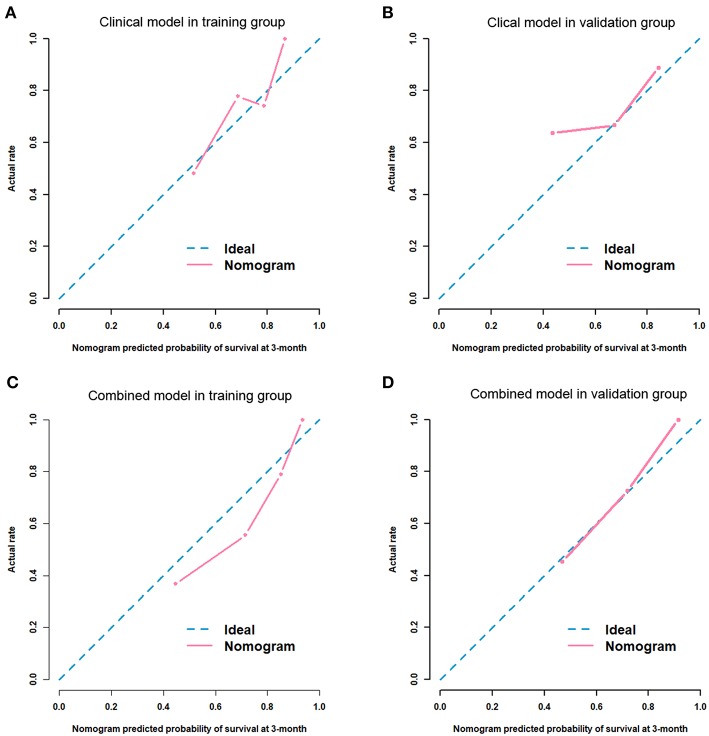
Calibration curves for the predictive performance for 3-month RFS. Clinical model in the training group (**A**, *p* = 0.105) and the validation group (**B**, *p* = 0.343). Combined model in the training group (**C**, *p* = 0.823) and the validation group (**D**, *p* = 0.329).

After univariate and multivariate analysis, N stage (HR [95% CI], 1.663 [1.041–2.659]; *p* = 0.033), M stage (HR [95% CI], 2.861 [1.114–7.352]; *p* = 0.029), and CA19-9 (HR [95% CI], 1.898 [1.024–3.520]; *p* = 0.042) were ultimately selected as clinical predictors of RFS (Table 3). The C-index for the clinical model was 0.673 in the training group and 0.667 in the validation group. The performance of the combined model was increased when the radiomics signature was added to the model, with a C-index of 0.791 in the training group and 0.779 in the validation group (Table 2). The nomograms for the clinical and combined models are shown in Figure 2. The performance for predicting 3-month RFS as shown by the area under the receiver operating characteristic curve was better with the combined model than with the clinical model for both groups (Figure 3). The calibration curves for the combined model demonstrated good agreement between the predicted and observed probabilities of progression at 3 months with *p*-values of 0.823 for the training group and 0.329 for the validation group (Figure 4).

**Table 3:** The univariate and multivariate analyses for clinical features in training group.

**Characteristics**	**HR**	**95% CI**	***p*-value**
**UNIVARIATE ANALYSIS**			
Age	1.000	(0.980, 1.021)	0.990
Sex	0.948	(0.573, 1.570)	0.948
BMI	0.965	(0.895, 1.040)	0.347
Length of obstruction	0.983	(0.958, 1.010)	0.213
TB	1.000	(0.998, 1.002)	0.962
DB	1.000	(0.998, 1.003)	0.806
DB/TB ratio	1.747	(0.164, 18.626)	0.644
Pain	1.278	(0.853, 1.914)	0.234
T stage	1.251	(0.843, 1.857)	0.265
N stage	1.868	(1.238, 2.818)	0.003*
M stage	2.026	(1.194, 3.435)	0.009*
Liver metastasis	1.518	(0.858, 2.688)	0.152
Number of metastatic lesions	1.559	(1.131, 2.148)	0.007*
Ascites	1.602	(1.050, 2.444)	0.029*
Radiotherapy	1.489	(0.361, 6.146)	0.582
Chemotherapy	0.607	(0.276, 1.331)	0.213
ECOG score	1.096	(0.785, 1.529)	0.592
Prior PTBD	1.211	(0.706, 2.077)	0.487
CA19-9	2.442	(1.454, 4.102)	0.001*
CA125	2.230	(1.286, 3.865)	0.004*
CEA	1.410	(0.870, 2.287)	0.163
**MULTIVARIATE ANALYSIS**			
N stage	1.663	(1.041, 2.659)	0.033*
M stage	2.861	(1.114, 7.352)	0.029*
Number of metastatic lesions	0.666	(0.345, 1.285)	0.225
Ascites	1.328	(0.825, 2.139)	0.243
CA19-9	1.898	(1.024, 3.520)	0.042*
CA125	1.627	(0.877, 3.016)	0.123

* *Data are statistically significant with p < 0.05. HR, hazard ratio; CI, confidence interval; BMI, body mass index; TB, total bilirubin; DB, direct bilirubin; ECOG, Eastern Cooperative Oncology Group; PTBD, percutaneous transhepatic biliary drainage; CA, carbohydrate antigen; CEA, carcinoembryonic antigen*.

### Clinical Use

The risk score for progression to the endpoint was calculated with the following formula.

$$\begin{array}{lll} \text{risk score} & = & exp\;(\text{1.179075+0.931}\times \text{M+0.753}\\ \; & \; & \times \text{N+0.509}\times \text{CA19-9}+1.139\times \text{Rad-score}) \end{array}$$

**Figure 5:**
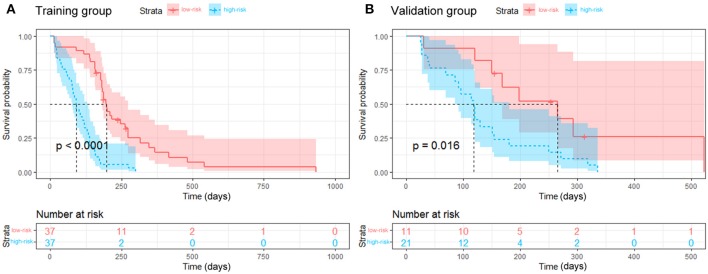
Kaplan-Meier curves for the stratified groups. The low-risk group had a longer RFS than the high-risk group in the training group (**A**, *p* < 0.001) and the validation group (**B**, *p* = 0.016).

The median risk score for progression in the training group (score = 1.264) was used to divide patients into high- (score ≥1.264) and low-risk (score < 1.264) groups. Kaplan-Meier curves and the log-rank test indicated significant differences in RFS between the high- and low-risk groups (median RFS: 90 days vs. 198 days, *p* < 0.001 for the training group; and median RFS: 118 days vs. 265 days, *p* = 0.016 for the validation group, Figure 5). The risk score also showed satisfactory stratification ability when adjusting to the different subgroups (all *p* < 0.05, Figure E4). As shown in Figure E5, the decision curve analysis for the individualized nomograms shows the overall net benefit in predicting RFS for the combined model was not inferior to the clinical model, the treat-all-patients scheme, and the treat-none scheme if the threshold probability of a patient was >51.0%.

## Discussion

Although irradiation stents have been applied to manage malignant intraluminal obstructive diseases (11, 12, 33–38), it is necessary to optimize the selection of appropriate patients for personalized treatment. In this study, a CT radiomics signature was combined with clinical features to establish an objective, preprocedural, and non-invasive model to select appropriate patients with UPC-MBO for irradiation stent placement. The combined model performed better than the clinical model.

With the combined nomogram, the 3-, 6-, and 12-month RFS probabilities can be calculated for each individual undergoing irradiation stent placement. With the risk score formula, each individual can be grouped into a low- or high-risk group. Two actual patients were classified using the combined model, as shown in Appendix E4 and Table E4, which demonstrated accurate prediction. “Patient A” with an RFS of 347 days had predicted 3-, 6-, and 12-month RFS probabilities of 0.85, 0.55, and 0.10, respectively, and was grouped into the low-risk group “Patient B” with an RFS of 129 days had predicted 3-, 6-, and 12-month RFS probabilities of 0.65, 0.18 and < 0.01, respectively, and was grouped into the high-risk group. It seems useful for clinical decision making that “Patient A” should undergo irradiation stent placement, but “Patient B” should undergo alternative treatment.

For patients with UPC-MBO, few biomarkers or models with good discrimination have been reported for prognostic prediction. Clinical indexes, including the CA19-9 level and N and M stages, have been applied to develop a model to predict prognosis in this study. The clinical model had a moderate C-index for discrimination (0.673 and 0.667 in the training and validation groups, respectively), while the radiomics signature showed a better C-index (0.787 and 0.796 in the training and validation groups, respectively). This result indicated better predictive performance of radiomic biomarkers than of clinical biomarkers. Moreover, the combined model also performed well with C-indexes of 0.791 and 0.779 in the training and validation groups, respectively. The reason may be that radiomics features from the tumor can provide more information on the cancer phenotype and the tumor microenvironment (39, 40), but clinical characteristics are limited.

As shown in Figure E4, regardless of which subgroup the patient was included in, he or she had a longer RFS in the low-risk group than in the high-risk group. Male sex, age older than 65 years, and an abnormal carcinoembryonic antigen level seemed to have less influence on RFS. Recently, researchers have been interested in the role of CA125 in pancreatic cancer (41). Positive CA125 levels may indicate tumor-associated Treg enrichment, which promotes tumor cell escape from the immune system (42). A high CA125 level is also associated with a high metabolic tumor burden (43) and poor prognosis (44–46). Although the CA125 level was a potential risk factor for RFS in the univariate analysis, this factor was not ultimately included in the predictive model developed with multivariate analysis (HR [95%]: 1.627 [0.877, 3.016]; *p* = 0.123). The prognostic importance of CA125 in pancreatic cancer should be further evaluated.

Imaging-based texture analysis is used to quantify intratumoral heterogeneity in patients with pancreatic cancer (21, 47, 48). Sandrasegaran et al. (19) demonstrated that contrast-enhanced CT-based radiomics features were associated with survival among patients with UPC, but only two-dimensional texture features from axial slices with maximum tumor dimensions were analyzed rather than features from multiple sections through the whole tumor. Cassinotto et al. (22) and Attiyeh et al. (23) evaluated only CT texture features in patients with surgically resectable pancreatic cancer. In our study, comprehensive radiomics features included intensity, shape, texture, and wavelet features that covered one-, two- and three-dimensional features in both the arterial and portal-venous phases. The radiomics signature based on both phases had good discrimination. In the arterial phase, the C-indexes were 0.735 and 0.719 in the training and validation groups, respectively. In the portal-venous phase, the C-indexes were 0.768 and 0.788 in the training and validation groups, respectively. Currently, few studies have explained the biological mechanisms of radiomics features for predicting treatment outcomes. However, this fact does not compromise the effectiveness and robustness of the proposed model for prognostic prediction.

This study has several limitations. First, as shown in Table E5, this study was a retrospective study with a small population. Second, evaluation of data from several independent centers for external validation is needed; however, this study was developed based on a limited sample. Third, the model was mainly used to choose appropriate patients for irradiation stent placement but was less able to predict the prognosis of patients who underwent placement of other stents or drainage mechanisms. Therefore, additional trials with large samples are needed to prospectively validate the findings in several independent centers. Radiogenomics-based studies are proposed for personalized treatment with radiotherapy or irradiation-related interventions for patients with pancreatic cancer.

## Conclusions

In conclusion, the proposed model based on radiomics had good performance for RFS prediction in patients with UPC-MBO who underwent irradiation stent placement. Patients with slow progression should consider undergoing irradiation stent placement for a longer RFS. With further sufficient validation and future clinical trials, this model might be an important tool for clinical decision making in interventional oncology.

## Data Availability Statement

The datasets generated for this study are available on request to the corresponding author.

## Ethics Statement

This study was approved by the institutional review boards at all participating centers, including IEC for Clinical Research of Zhongda Hospital, Southeast University, IEC for Clinical Research of Yunnan Tumor Hospital, IEC for Clinical Research of Lishui Central Hospital, and IEC for Clinical Research of Anhui Provincial Hospital.

## Author Contributions

G-JT, H-FZ, Y-QH, JL, J-HG, and H-DZ contributed to the study concept and design. MH, J-SJ, W-FL, and G-YZ contributed to perform stent placement. JL, LC, and Z-CJ contributed to the acquisition of clinical data. J-WW contributed to the statistical analysis. H-FZ, Y-QH, H-DZ, and J-HG contributed to data interpretation. H-FZ wrote the first draft of the manuscript. Y-QH wrote the sections of the manuscript. G-JT, JT, H-DZ, and J-HG supervised and oversaw the study. All authors contributed to manuscript revision, read, and approved the submitted version.

### Conflict of Interest

The authors declare that the research was conducted in the absence of any commercial or financial relationships that could be construed as a potential conflict of interest.
