# Supplementary material for: Radiomics Facilitates Candidate Selection for Irradiation Stents Among Patients With Unresectable Pancreatic Cancer
Source: Front Oncol. 2019 Sep 27;9:973. doi: 10.3389/fonc.2019.00973 (PMC6776612; doi:10.3389/fonc.2019.00973)
Supplement: Supplementary file 1 [file Data_Sheet_1.docx]

Supplementary Material

# Appendix E1: Sample size consideration

In our study, a Cox proportional hazards regression model was used to predict the prognosis of RFS. In terms of the training sample size, a rule of thumb was previously proposed that at least ten events-per-predictor is required to produce reasonably stable estimates (1, 2). Vittinghoff and colleagues suggested that the rule of ten events-per-predictor in logistic and Cox regression models could range from five to nine (3). In our study, three predictors in the clinical model indicated a minimum size of 15 to 30, eight features in the arterial phase radiomics signature indicated a minimum size of 40 to 80, six features in the portal-venous phase radiomics signature indicated a minimum size of 30 to 60, and four predictors in the combined model indicated a minimum size of 20 to 40. Therefore, 74 patients with 70 events in the training group were considered adequate.

In terms of the validation sample size, a power calculation to estimate the sample size for Cox regression was performed with PASS 15 (NCSS, LLC., Kaysville, Utah, USA) (4, 5). According to the training group, a Cox regression model for the Rad-score had a log hazard ratio on a covariate with a standard deviation of 0.7745, and the regression coefficient was 0.8932 with 90% power at a 0.05000 significance level. The sample size of events was 22. Therefore, 32 patients with 29 events in the validation group were considered adequate.

Gillies et al. noticed that although larger data sets provide more power, radiomics can be performed with as few as 100 patients (6). Moreover, ten-fold cross validation was used in our study to optimize the regression model to select the most reliable model.

# Appendix E2: CT acquisition protocols and image preprocessing

The CT acquisition protocols were similar in these four centers. Enhanced CT was examined using one of the two followed multidetector row CT scanners: SOMATOM Definition AS+ (Siemens Medical systems) and SOMATOM Sensation 64 (Siemens Medical systems). Routine enhanced CT scan was performed including arterial and portal-venous phases at 20-30s and 40-50 s, respectively, after injecting contrast material (300-370 mgI/ml, 1.5 ml/kg, 3.0~ 3.5 ml/s). Scanning parameters were as follows: tube voltage 120kVp, tube current 100-500mA, detector collimation 128×0.6mm or 64×0.625mm, field of view 500mm×500mm, pixel size 512×512, rotation time 0.50s, slice interval 5mm, and slice thickness 5 mm.

All images were normalized by centering it at the mean with standard deviation (the intensity of CT image was scale to 0-1) to reduce the impact of image heterogeneity in four centers. Normalization is based on all gray values in the image according to the following equation:

$$\boldsymbol{x}^{\boldsymbol{'}}\boldsymbol{=}\frac{\boldsymbol{(x-}\boldsymbol{\mu}_{\boldsymbol{x}}\boldsymbol{)}}{\boldsymbol{\sigma}_{\boldsymbol{x}}}$$

$x$ and $x^{'}$ are the original and normalized intensity, respectively. $\mu_{x}$ and $\sigma_{x}$ are the mean and standard deviation of the image intensity, respectively.

# Appendix E3: CT-based radiomics features

The specific feature definitions used in this study are shown in Table E1. Different types of radiomics features, including eight shape features, 17 tumour intensity features, and 51 texture and wavelet features with different wavelet decompositions of the original images, were included. The shape features were calculated based on the shape of the tumour, such as the surface area and volume. The tumour intensity features were calculated with first-order statistics, which describe the distribution of voxel intensities in CT images. These two types of features offer information that is associated with the grey-level distribution of an image, but they offer no information concerning the relative position of the various grey levels in an image. Therefore, texture features that describe the distribution of voxel intensities and are calculated based on four textural matrices, namely, grey-level cooccurrence matrix (GLCM), grey-level run-length matrix (GLRLM), grey-level size-zone matrix (GLSZM), and neighbourhood grey-tone difference matrix (NGTDM), were also included in this study. We applied undecimated three-dimensional wavelet transformation to decompose the original image into eight parts. L and H indicate low- and high-pass functions, respectively. The wavelet decompositions of the original image X were labelled as XLLL, XLLH, XLHL, XLHH, XHLL, XHLH, XHLH, and XHHH. For example, XLLH represents the image filtered with a low-pass function in the x- and y-directions and a high-pass function in the z-direction, as the following formula.

$$\boldsymbol{X}_{\boldsymbol{LLH}}\left( \boldsymbol{i}\mathbf{,}\boldsymbol{j}\mathbf{,}\boldsymbol{k} \right)\boldsymbol{=}\sum_{\boldsymbol{p}\mathbf{=1}}^{\boldsymbol{Nl}} \sum_{\boldsymbol{q}\mathbf{=1}}^{\boldsymbol{Nl}} \sum_{\boldsymbol{r}\mathbf{=1}}^{\boldsymbol{Nh}} \boldsymbol{L}\mathbf{(}\boldsymbol{p}\mathbf{)}\boldsymbol{L}\mathbf{(}\boldsymbol{q}\mathbf{)}\boldsymbol{H}\mathbf{(}\boldsymbol{r}\mathbf{)}\boldsymbol{X}\mathbf{(}\boldsymbol{i}\mathbf{+}\boldsymbol{p}\mathbf{,}\boldsymbol{j}\mathbf{+}\boldsymbol{q}\mathbf{,}\boldsymbol{k}\mathbf{+}\boldsymbol{r}\mathbf{)}$$

In this formula, *Nl* is the length of filter L, and *Nh* is the length of filter H.

After Pearson’s correlation analysis, a total of 61 features from the arterial phase and 49 features from the portal-venous phase were identified as independent (Table E2). According to the LASSO-Cox model eight features from the arterial phase and six features from the portal-venous phase were identified that most efficient for predicting RFS, and Table E3 showed no significant difference between the training and validation groups (all *p* > 0.05).

# Appendix E4: Actual cases by using the combined model of RFS predictive nomogram and risk score formula

Four biomarkers were selected for the combined model, including three categorical biomarkers (CA 19-9 level, N stage, and M stage) and one continuous biomarker (Rad-score). The values of each biomarker were quantified. A CA19-9 level less than 1000 U/ml was recorded as 0 and as 1 for levels of 1000 U/ml or greater. N stages of 0, 1, or 2 were recorded 0, 1, or 2, respectively. M stages of 0 or 1 were recorded 0 or 1, respectively. The Rad-score ranged from –0.8 to 4.8. To use the combined nomogram, the 3-, 6-, and 12-month RFS probabilities could be calculated according to the points of the Rad-score, CA19-9 level, and N and M stages. For example, “Patient A” had a total of 3.3 points with a Rad-score of – 0.3 (1.0 points), a CA19-9 level < 1000 U/ml (0 points), N1 (1.0 points) and M1 (1.3 points); the 3-, 6-, and 12-month RFS probabilities were approximately 0.85, 0.55, and 0.10, respectively. “Patient B” had a total of 4.8 points with a Rad-score of 0.1, a CA19-9 level > 1000 U/ml, N1 and M1, and the 3-, 6- and 12-month RFS probabilities were approximately 0.65, 0.18 and < 0.01, respectively. According to the formula for the risk score for progression:

$$\mathbf{risk score =}\boldsymbol{exp}\boldsymbol{(-1.179075+0.931\times M+0.753\times N+0.509\times CA19-9+1.139\times Rad-score)}$$

“Patient A” was in the low-risk group with a risk score of 1.14 (< 1.264), and “Patient B” was in the high-risk group with a risk score of 3.04 (> 1.264). Corresponding to the actual cases, “Patient A” had an RFS of 347 days (11 months), and “Patient B” had an RFS of 129 days (4 months) (Table E4).

# Appendix E5: Supplementary Tables and Figures

## Supplementary Tables

**Table E1. Non-texture and texture features used in this study.**

| **Non-Texture Features (n=25)** | | | | | |  |  |
| --- | --- | --- | --- | --- | --- | --- | --- |
| ***Shape and Size Features (n=8)*** | | | | | |  |  |
| **Reference** | | | | **Feature** | |  |  |
| **——** | | | | Compactness1  Compactness2 | |  |  |
|  |  |  |  | Maximum 3D diameter  Spherical disproportion  Sphericity  Surface area  Surface to volume ratio  Volume | |  |  |
| ***First Order Statistic Features (n=17)*** | | | | |  |  |  |
| **Reference** | | **Feature** | | |  |  |  |
| **——** | | Energy  Entropy | | |  |  |  |
|  |  | Kurtosis  Maximum  Mean  Mean absolute deviation  Median  Minimum  Range  Root mean square  Skewness  Standard deviation  Uniformity  Variance  Sum  Entropy after normalization  Uniformity after normalization | | |  |  |  |
| **Texture Features (n=51)** | | | | | | |  |
| ***GLCM Features (n=22)*** | | | | | | |  |
| **Reference** | | **Feature** | | | | |  |
| **Haralick et al. 1973 (7)** | | Autocorrelation  Cluster prominence  Cluster shade  Cluster tendency  Contrast  Correlation  Difference entropy  Dissimilarity  Energy  Entropy  Homogeneity 1  Homogeneity 2  Information measure of correlation 1  Information measure of correlation 2  Inverse difference moment normalized  Inverse difference normalized  Inverse variance  Maximum probability  Sum average  Sum entropy  Sum variance  Covariance | | | | |  |
| ***GLRLM Features (n=11)*** | | | | | | |  |
| **Reference** | **Feature** | | | | | |  |
| **Galloway 1975 (8)** | Short Run Emphasis (SRE) | | | | | |  |
|  | Long Run Emphasis (LRE) | | | | | |  |
|  | Grey Level Non-Uniformity (GLN) | | | | | |  |
|  | Run Length Non-Uniformity (RLN) | | | | | |  |
|  | Run Percentage (RP) | | | | | |  |
| **Chu et al. 1990 (9)** | Low Grey Level Run Emphasis (LGLRE) | | | | | |  |
|  | High Grey Level Run Emphasis (HGLRE) | | | | | |  |
| **Dasarathy and Holder 1991 (10)** | Short Run Low Grey Level Emphasis (SRLGLE) | | | | | |  |
|  | Short Run High Grey Level Emphasis (SRHGLE) | | | | | |  |
|  | Long Run Low Grey Level Emphasis (LRLGLE) | | | | | |  |
|  | Long Run High Grey Level Emphasis (LRHGLE) | | | | | |  |
| ***GLSZM Features (n=13)*** | | | | | | | |
| **Reference** | | | **Feature** | | | | |
| **Galloway 1975 (8)** | | | Small Zone Emphasis (SZE) | | | | |
|  | | | Large Zone Emphasis (LZE) | | | | |
|  | | | Grey-Level Non-uniformity (GLN) | | | | |
|  | | | Zone-Size Non-uniformity (ZSN) | | | | |
|  | | | Zone Percentage (ZP) | | | | |
| **Chu et al. 1990 (9)** | | | Low Grey-Level Zone Emphasis (LGZE) | | | | |
|  | | | High Grey-Level Zone Emphasis (HGRE) | | | | |
| **Dasarathy and Holder 1991 (10)** | | | Small Zone Low Grey-Level Emphasis (SZLGE) | | | | |
|  | | | Small Zone High Grey-Level Emphasis (SZHGE) | | | | |
|  | | | Large Zone Low Grey-Level Emphasis (LZLGE) | | | | |
|  | | | Large Zone High Grey-Level Emphasis (LZHGE) | | | | |
| **Thibault et al. 2009 (11)** | | | Grey-Level Variance (GLV) | | | | |
|  | | | Zone-Size Variance (ZSV) | | | | |
| ***NGTDM Features (n=5)*** | | | | | | | |
| **Reference** | | | **Feature** | | | | |
| **Amadasun and King 1989 (12)** | | | Coarseness | | | | |
|  | | | Contrast | | | | |
|  | | | Busyness | | | | |
|  | | | Complexity | | | | |
|  | | | Strength | | | | |

**Table E2. Independent feature for plain in arterial and portal-venous phases.**

| **AP plain (n = 61)** | **PP plain (n = 49)** |
| --- | --- |
| ori_fos_minimum | ori_fos_energy |
| ori_glcm_cluster_tendency | ori_fos_minimum |
| ori_glcm_IMC2 | ori_fos_uniformity |
| ori_glrlm_RP | ori_glcm_homogeneity2 |
| ori_glrlm_SRHGLE | ori_glrlm_SRHGLE |
| ori_glszm_SZSE | ori_glszm_SZLGE |
| ori_glszm_SZLGE | ori_glszm_LZHGE |
| ori_glszm_ZSP | ori_glszm_ZSV |
| ori_glszm_ZSV | Coif1_fos_uniformity |
| Coif1_glcm_dissimilarity | Coif1_glcm_homogeneity2 |
| Coif1_glrlm_RP | Coif1_glcm_inverse_variance |
| Coif1_glrlm_SRHGLE | Coif1_glrlm_SRHGLE |
| Coif1_glrlm_LRHGLE | Coif1_glrlm_LRHGLE |
| Coif1_glszm_LGLZE | Coif1_glszm_LGLZE |
| Coif1_glszm_SZLGE | Coif1_glszm_SZHGE |
| Coif1_glszm_SZHGE | Coif1_glszm_GLV |
| Coif1_glszm_LZHGE | Coif1_glszm_ZSV |
| Coif1_glszm_GLV | Coif2_fos_range |
| Coif1_glszm_ZSV | Coif2_glcm_cluster_shade |
| Coif2_fos_maximum | Coif2_glcm_cluster_tendency |
| Coif2_fos_median | Coif2_glcm_contrast |
| Coif2_fos_entropy_p | Coif2_glcm_correlation |
| Coif2_fos_uniformity_p | Coif2_glcm_sum_variance |
| Coif2_glcm_cluster_prominence | Coif2_glrlm_SRE |
| Coif2_glcm_cluster_shade | Coif2_glrlm_RLN |
| Coif2_glcm_cluster_tendency | Coif2_glrlm_LGLRE |
| Coif2_glcm_correlation | Coif2_glrlm_SRLGLE |
| Coif2_glcm_IMC1 | Coif2_glrlm_SRHGLE |
| Coif2_glcm_sum_variance | Coif2_ngtdm_coarseness |
| Coif2_glcm_covariance | Coif2_ngtdm_contrast |
| Coif2_glrlm_SRE | Coif2_glszm_SZSE |
| Coif2_glrlm_LGLRE | Coif2_glszm_HGLZE |
| Coif2_glrlm_SRLGLE | Coif2_glszm_SZLGE |
| Coif2_glrlm_SRHGLE | Coif3_fos_uniformity_p |
| Coif2_glszm_SZSE | Coif4_glcm_correlation |
| Coif2_glszm_LGLZE | Coif4_glcm_IMC1 |
| Coif2_glszm_HGLZE | Coif4_glcm_inverse_variance |
| Coif2_glszm_ZSP | Coif5_glcm_correlation |
| Coif3_glcm_autocorrelation | Coif5_ngtdm_complexity |
| Coif3_glcm_covariance | Coif5_glszm_HGLZE |
| Coif3_glrlm_HGLRE | Coif5_glszm_SZLGE |
| Coif3_ngtdm_strength | Coif6_glcm_covariance |
| Coif4_glszm_LGLZE | Coif7_fos_range |
| Coif4_glszm_HGLZE | Coif7_glcm_correlation |
| Coif4_glszm_LZLGE | Coif7_glszm_LGLZE |
| Coif5_fos_maximum | Coif7_glszm_HGLZE |
| Coif5_fos_mean | Coif8_glrlm_GLN |
| Coif5_glcm_IMC1 | Coif8_glrlm_HGLRE |
| Coif5_glszm_SZSE | Coif8_glszm_HGLZE |
| Coif5_glszm_LGLZE |  |
| Coif6_glcm_covariance |  |
| Coif7_glcm_correlation |  |
| Coif7_glcm_inverse_variance |  |
| Coif7_glcm_covariance |  |
| Coif7_glszm_HGLZE |  |
| Coif8_fos_minimum |  |
| Coif8_glcm_correlation |  |
| Coif8_glcm_IMC1 |  |
| Coif8_glcm_IMC2 |  |
| Coif8_glrlm_HGLRE |  |
| Coif8_ngtdm_complexity |  |

Abbreviations: AP, arterial phase; PP, portal-venous phase.

**Table E3. Radiomic biomarkers between the training and validation groups.**

| **Features** | **Training**  **(n = 74)** | **Validation**  **(n = 32)** | ***p* value** |
| --- | --- | --- | --- |
| **PP_ori_glszm_ZSV** | 122.48 | 74.43 | 0.402 |
| **PP_Coif1_fos_uniformity** | 2.39 E+8 | 1.32E+8 | 0.294 |
| **PP_Coif2_glrlm_SRHGLE** | 118.08 | 118.31 | 0.978 |
| **PP_Coif5_glcm_correlation** | 0.09 | 0.10 | 0.829 |
| **PP_Coif6_ngtdm_complexity** | 119.71 | 122.21 | 0.914 |
| **PP_Coif8_glszm_HGLZE** | 170.46 | 169.82 | 0.366 |
| **AP_Coif1_glszm_LZHGE** | 7.17E+10 | 5.50E+10 | 0.747 |
| **AP_Coif2_fos_median** | 108.66 | 98.84 | 0.098 |
| **AP_Coif5_glszm_SZSE** | 0.52 | 0.52 | 0.958 |
| **AP_Coif7_glcm_inverse_variance** | 0.33 | 0.33 | 0.918 |
| **AP_Coif8_fos_minimum** | 83.91 | 83.06 | 0.755 |
| **AP_Coif8_glcm_IMC2** | 0.19 | 0.18 | 0.947 |
| **AP_Coif8_glszm_LGLZE** | 0.01 | 0.01 | 0.338 |
| **AP_Coif8_glszm_HGLZE** | 170.15 | 169.73 | 0.513 |

Abbreviations: AP, arterial phase; PP, portal-venous phase.

**Table E4. Actual cases by using the combined model.**

| **Cases** | **Values of biomarkers** | | | | **Total points** | **RFS probability** | | | **Risk score for progress** | **Risk group** | **RFS (days)** |
| --- | --- | --- | --- | --- | --- | --- | --- | --- | --- | --- | --- |
|  | **N stage** | **M stage** | **CA 19-9** | **Rad-score** |  | **3-month** | **6-month** | **12-month** |  |  |  |
| Patient A | 1 | 1 | 0 | –0.3 | 3.3 | 0.85 | 0.55 | 0.10 | 1.14 | Low-risk | 347 |
| Patient B | 1 | 1 | 1 | 0.1 | 4.8 | 0.65 | 0.18 | < 0.01 | 3.04 | High-risk | 129 |

Abbreviations: CA, carbohydrate antigen; RFS, restenosis-free survival.

**Table E5. Patient characteristics in each center.**

| **Characteristics** | **Center 1 (n=7)** | **Center 2 (n=16)** | **Center 3 (n=57)** | **Center 4 (n=26)** |
| --- | --- | --- | --- | --- |
| **Age, mean ± SD, years** | 63.86 ± 10.57 | 68.81 ± 14.30 | 63.53 ± 11.02 | 68.77 ± 12.27 |
| **Sex, n (%)** |  |  |  |  |
| Male | 4 (57.1) | 7 (43.8) | 39 (68.4) | 19 (73.1) |
| Female | 3 (42.9) | 9 (56.3) | 18 (31.6) | 7 (26.9) |
| **BMI, mean ± SD, kg/m2** | 19.46 ± 2.92 | 20.76 ± 3.10 | 20.49 ± 3.29 | 20.99 ± 2.63 |
| **Length of obstruction, mean ± SD, mm** | 36.71 ± 5.22 | 39.38 ± 4.43 | 38.70 ± 10.94 | 34.62 ± 11.04 |
| **TB, mean ± SD, µmol/L** | 223.71 ± 138.09 | 121.82 ± 85.52 | 212.64 ± 154.70 | 153.22 ± 87.03 |
| **DB, mean ± SD, µmol/L** | 180.40 ± 110.25 | 93.78 ± 99.51 | 159.52 ± 109.57 | 112.04 ± 63.52 |
| **DB/TB ratio, mean ± SD** | 0.806 ± 0.036 | 0.759 ± 0.098 | 0.762 ± 0.113 | 0.734 ± 0.123 |
| **Pain, n (%)** |  |  |  |  |
| None | 3 (42.9) | 2 (12.5) | 10 (17.5) | 8 (30.8) |
| Mild | 2 (28.6) | 4 (25.0) | 43 (75.4) | 14 (53.8) |
| Moderate or severe | 2 (28.6) | 10 (62.5) | 4 (7.0) | 4 (15.4) |
| **T stage, n (%)** |  |  |  |  |
| 2 | 0 (0) | 0 (0) | 10 (17.5) | 0 (0) |
| 3 | 0 (0) | 0 (0) | 10 (17.5) | 1 (3.8) |
| 4 | 7 (100) | 16 (100) | 37 (64.9) | 25 (96.2) |
| **N stage, n (%)** |  |  |  |  |
| 0 | 1 (14.3) | 4 (25.0) | 18 (31.6) | 3 (11.5) |
| 1 | 5 (71.4) | 7 (43.8) | 39 (68.4) | 17 (65.4) |
| 2 | 1 (14.3) | 5 (31.3) | 0 (0) | 6 (23.1) |
| **M stage, n (%)** |  |  |  |  |
| 0 | 2 (28.6) | 9 (56.3) | 42 (73.7) | 15 (57.7) |
| 1 | 5 (71.4) | 7 (43.8) | 15 (26.3) | 11 (42.3) |
| **Liver metastasis, n (%)** |  |  |  |  |
| No | 3 (42.9) | 13 (81.3) | 45 (78.9) | 15 (57.7) |
| Yes | 4 (57.1) | 3 (18.8) | 12 (21.1) | 11 (42.3) |
| **Number of metastatic lesions, n (%)** |  |  |  |  |
| 0 | 2 (28.6) | 10 (62.5) | 43 (75.4) | 13 (50.0) |
| 1 | 2 (28.6) | 0 (0) | 3 (5.3) | 7 (26.9) |
| ≥ 2 | 3 (42.9) | 6 (37.5) | 11 (19.3) | 6 (23.1) |
| **Ascites level, n (%)** |  |  |  |  |
| None | 6 (85.7) | 12 (75.0) | 50 (87.7) | 17 (65.4) |
| Mild | 0 (75.0) | 2 (12.5) | 6 (10.5) | 6 (23.1) |
| Moderate or severe | 1 (14.1) | 2 (12.5) | 1 (1.8) | 3 (11.5) |
| **Radiotherapy, n (%)** |  |  |  |  |
| No | 7 (100) | 15 (93.8) | 55 (96.5) | 24 (92.3) |
| Yes | 0 (0) | 1 (6.3) | 2 (3.5) | 2 (7.7) |
| **Chemotherapy, n (%)** |  |  |  |  |
| No | 4 (57.1) | 16 (100) | 48 (84.2) | 23 (88.5) |
| Yes | 3 (42.9) | 0 (0) | 9 (15.8) | 3 (11.5) |
| **ECOG score, n (%)** |  |  |  |  |
| 0 | 0 (0) | 0 (0) | 2 (3.5) | 1 (3.8) |
| 1 | 1 (14.3) | 1 (6.3) | 6 (10.5) | 3 (11.5) |
| 2 | 4 (57.1) | 9 (56.3) | 33 (57.9) | 14 (53.8) |
| 3 | 2 (28.6) | 6 (37.5) | 16 (28.1) | 8 (30.8) |
| **Prior PTBD, n (%)** |  |  |  |  |
| No | 1 (14.3) | 1 (6.3) | 17 (29.8) | 12 (46.2) |
| Yes | 6 (85.7) | 15 (93.8) | 40 (70.2) | 14 (53.8) |
| **CA19-9, n (%)** |  |  |  |  |
| < 1000 U/ml | 3 (42.9) | 5 (31.3) | 35 (61.4) | 14 (53.8) |
| ≥ 1000 U/ml | 4 (57.1) | 11 (68.8) | 22 (38.6) | 12 (46.2) |
| **CA125, n (%)** |  |  |  |  |
| < 35 U/ml | 4 (57.1) | 2 (12.5) | 17 (29.8) | 10 (38.5) |
| ≥ 35 U/ml | 3 (42.9) | 14 (87.5) | 40 (70.2) | 16 (61.5) |
| **CEA, n (%)** |  |  |  |  |
| < 5 ng/ml | 4 (57.1) | 4 (25.0) | 23 (40.4) | 10 (38.7) |
| ≥ 5 ng/ml | 3 (42.9) | 12 (75.0) | 34 (59.6) | 16 (61.5) |

## Supplementary Figures


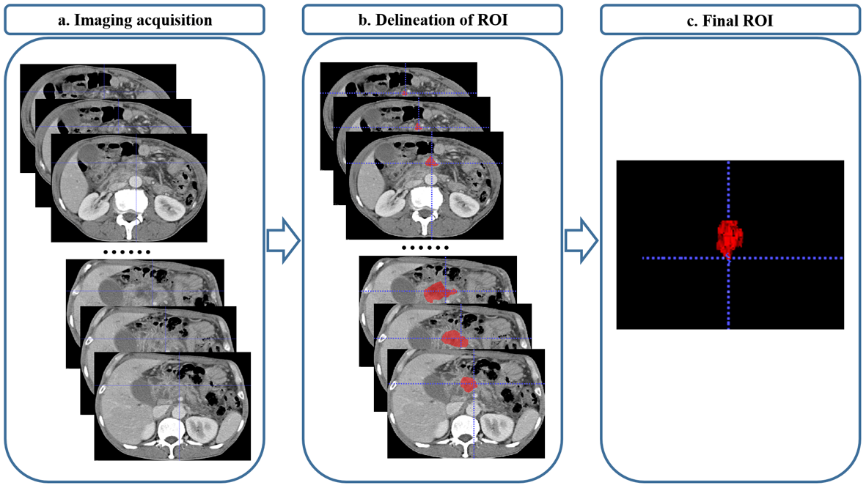


Figure E1. Flowchart of the ROI segamentation.


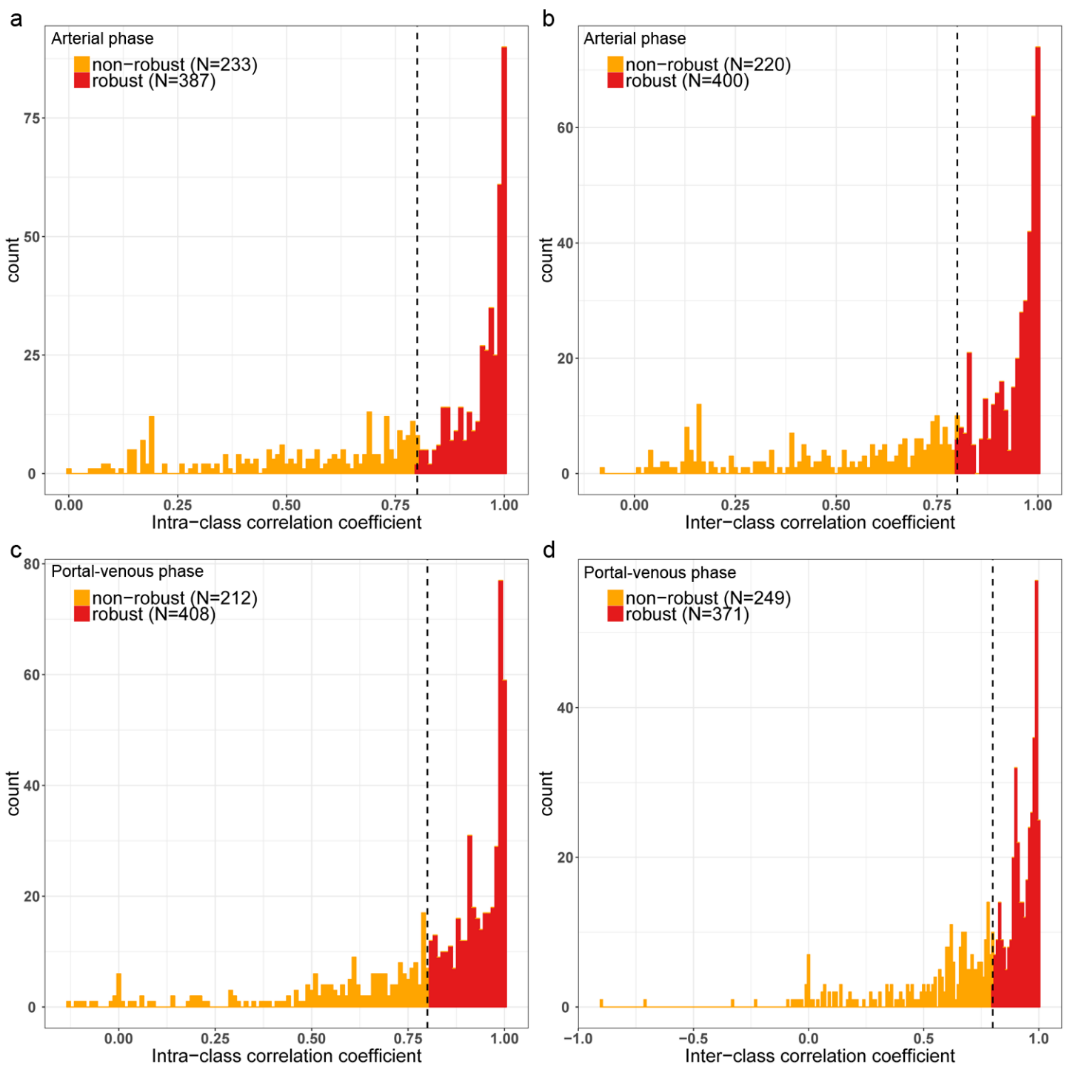


**Figure E2.** The intra- and inter- class correlation coefficient analyses for radiomic features. The stability of features was detected with a threshold of 0.8 in arterial phase (a; b) and portal-venous phase (c; d).


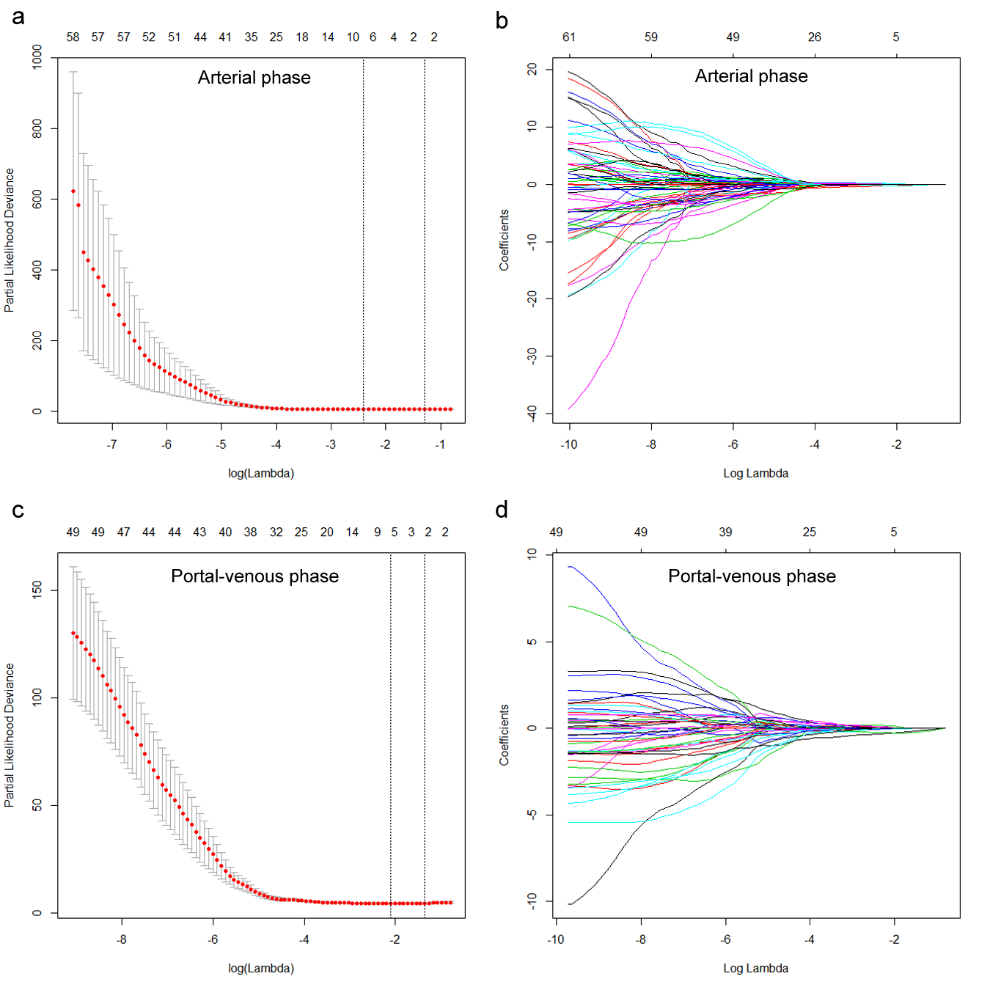


**Figure E3.** The least absolute shrinkage and selection operator (LASSO)-Cox model for selecting radiomic biomarkers. Turning parameter (lambda) selection in the LASSO model (a; c). The solid grey vertical lines represent the partial likelihood deviance ± standard error (SE). The dotted vertical lines are drawn at the optimal values by minimum criteria and 1-SE criteria. It plotted the partial likelihood deviance versus log (lambda), where lambda is the tuning parameter. The optimal lambda = 0.09 with log (lambda) = -2.4 was chosen in arterial phase (a), and the optimal lambda = 0.12 with log (lambda) = -2.1 was chosen in portal-venous phase (c). LASSO coefficient profiles of the radiomic features (b; d). A coefficient profile plot was produced against the log (lambda) sequence. The optimal lambda resulted in eight nonzero coefficients in arterial phase (b) and six nonzero coefficients in portal-venous phase (d).


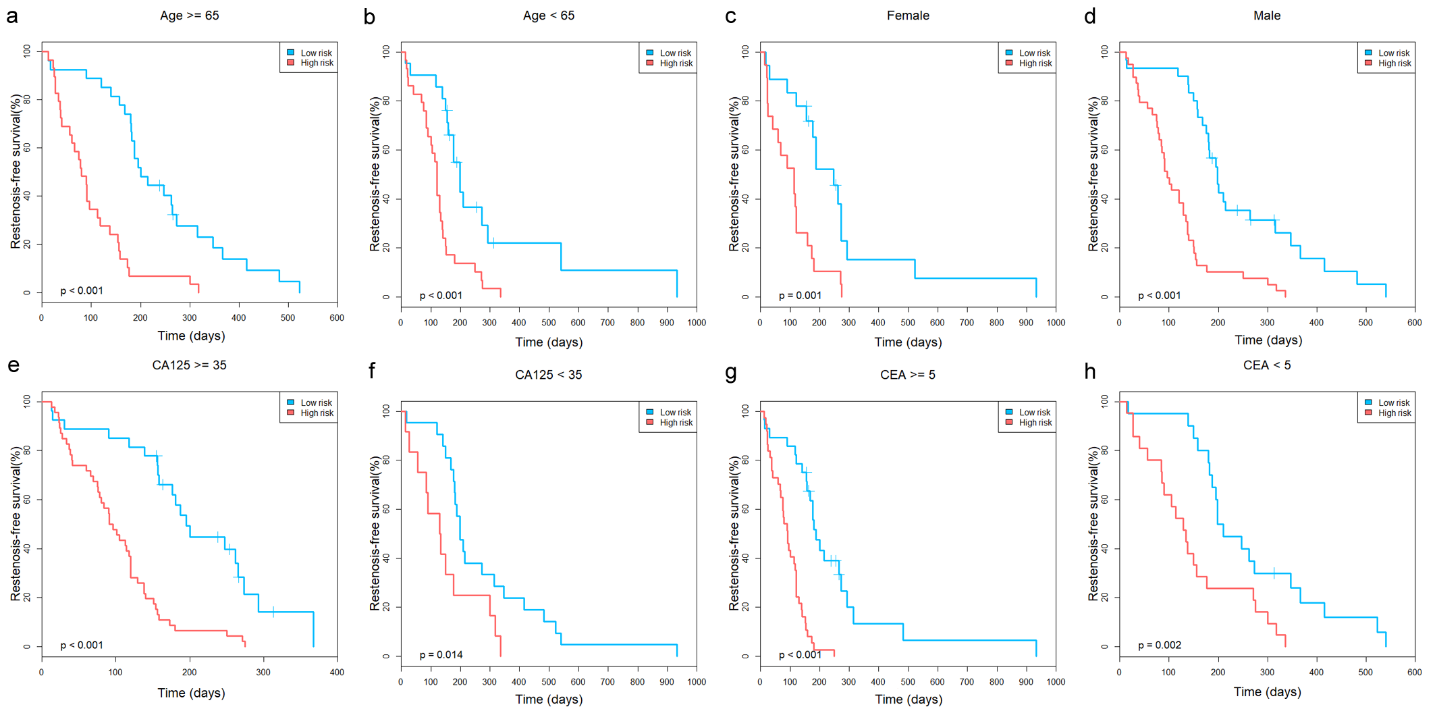


**Figure E4.** Kaplan-Meier curves for subgroups compared between low-risk group and high-risk group. (a) Subgroup of age **≥** 65 years (*p* < 0.001). (b) Subgroup of age **<** 65 years (*p* < 0.001). (c) Female subgroup (*p* < 0.001). (d) Male subgroup (*p* < 0.001). (e) Subgroup of carbohydrate antigen (CA)125 **≥** 35 U/ml (*p* < 0.001). (f) Subgroup of CA125 **<** 35 U/ml (*p* = 0.014). (g) Subgroup of carcinoembryonic antigen (CEA) **≥** 5 ng/ml (*p* < 0.001). (h) Subgroup of CEA **<** 5 ng/ml (*p* = 0.002).


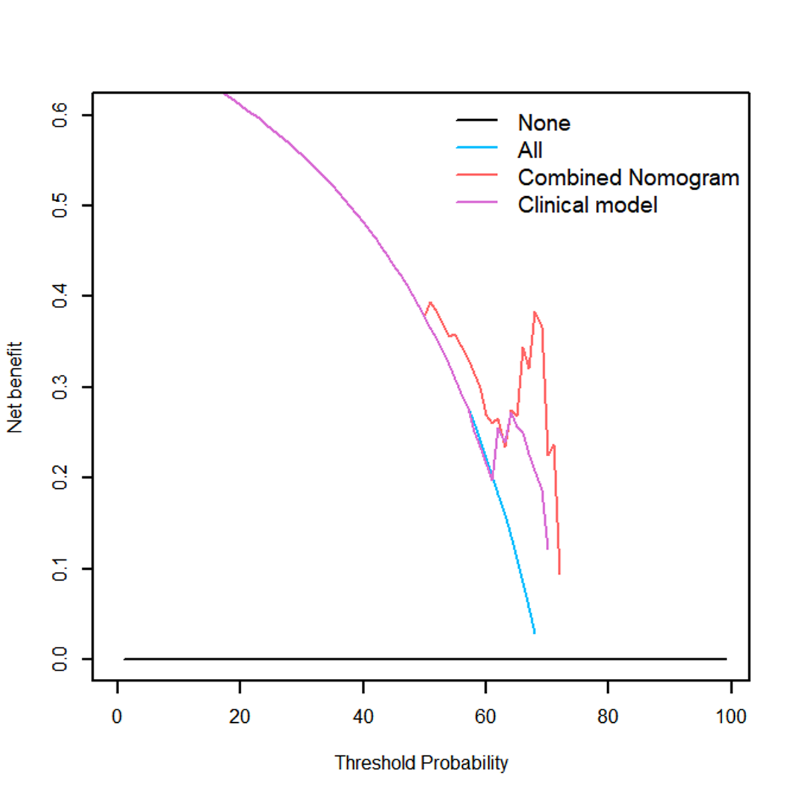


**Figure E5.** Decision curve analysis for comparing prognostic model with (combined model) and without (clinical model) integrating the radiomics signature. The net benefit according to the relative harm of forgoing treatment compared with the negative consequences of an unnecessary treatment was calculated by subtracting the proportion of all patients who were false positives from the proportion who were true positives.

**Supplementary References**

1. Concato J, Peduzzi P, Holford TR, Feinstein AR. Importance of events per independent variable in proportional hazards analysis. I. Background, goals, and general strategy. *J Clin Epidemiol*. (1995) 48: 1495-501.

2. Peduzzi P, Concato J, Feinstein AR, Holford TR. Importance of events per independent variable in proportional hazards regression analysis II. Accuracy and precision of regression estimates. *J Clin Epidemiol*. (1995) 48: 1503-10. doi:10.1016/0895-4356(95)00048-8

3. Vittinghoff E, McCulloch CE. Relaxing the rule of ten events per variable in logistic and Cox regression. *Am J Epidemiol*. (2007) 165: 710-8. doi:10.1093/aje/kwk052

4. Hsieh FY, Lavori PW. Sample-size calculations for the Cox proportional hazards regression model with nonbinary covariates. *Control Clin Trials*. (2000) 21: 552-60.

5. Schoenfeld DA. Sample-size formula for the proportional-hazards regression model. *Biometrics*. (1983) 39: 499-503.

6. Gillies RJ, Kinahan PE, Hricak H. Radiomics: images are more than pictures, they are data. *Radiology*. (2016) 278: 563-77. doi:10.1148/radiol.2015151169

7. Haralick RM, Shanmugam K, Dinstein I. Textural features for image classification. *IEEE Transactions on Systems, Man and Cybernetics*. (1973) SMC-3; SMC-3: 610-21. doi:10.1109/TSMC.1973.4309314

8. Galloway MM. Texture analysis using gray level run lengths. *Computer Graphics and Image Processing*. (1975) 4: 172-9. doi:https://doi.org/10.1016/S0146-664X(75)80008-6

9. Chu A, Sehgal CM, Greenleaf JF. Use of gray value distribution of run lengths for texture analysis. *Pattern Recogn Lett*. (1990) 11: 415-9. doi:https://doi.org/10.1016/0167-8655(90)90112-F

10. Dasarathy BV, Holder EB. Image characterizations based on joint gray level—run length distributions. *Pattern Recogn Lett*. (1991) 12: 497-502. doi:https://doi.org/10.1016/0167-8655(91)80014-2

11. Thibault G, Fertil B, Navarro C, Pereira S, Cau P, Levy N, et al. Texture indexes and gray level size zone matrix application to cell nuclei classification. In: *10th International Conference on Pattern Recognition and Information Processing*., (2009)

12. Amadasun M, King R. Textural features corresponding to textural properties. *IEEE Transactions on Systems, Man and Cybernetics*. (1989) 19; 19: 1264-74. doi:10.1109/21.44046
